# Sanitary Measures in Egypt

**Published:** 1847-11

**Authors:** 


*Sanitary Measures in Egypt.*—The Egyptian government, to give the fullest effect to quarantine regulations, has ordered the number of European and Arab physicians to be doubled throughout the country. Until the present time, there was one Arab and one European physician to fifty villages. Now there are to be four for the same locality, and this number may be increased when they are procurable. At the school of medicine at Cairo, the number of pupils has been doubled, as also the class of inferior medical officers. Such a demand for medical men in Egypt surely offers an opportunity to some of our over-numerous body to exercise the healing art in that country, and in a more profitable manner than they can hope to do at home, under the tender mercies of Boards of Health and Guardians, who consider five shillings per diem ample pay for any amount of toil, and any risk of life.—*Ibid.*

